# Functional analysis of auxin derived from a symbiotic mycobiont

**DOI:** 10.3389/fpls.2023.1216680

**Published:** 2023-09-08

**Authors:** Cheng-Yen Chen, Poonguzhali Selvaraj, Naweed I. Naqvi

**Affiliations:** ^1^ Fungal Patho-Biology, Temasek Life Sciences Laboratory, Singapore, Singapore; ^2^ Department of Biological Sciences, National University of Singapore, Singapore, Singapore

**Keywords:** auxin, indole-3-acetic acid, *Penicillium citrinum*, phytohormone, plant growth, root development, symbiosis

## Abstract

The biosynthesis of auxin or indole-3-acetic acid by microorganisms has a major impact on plant–microbe interactions. Several beneficial microbiota are known to produce auxin, which largely influences root development and growth in the host plants. Akin to findings in rhizobacteria, recent studies have confirmed the production of auxin by plant growth-promoting fungi too. Here, we show that *Penicillium citrinum* isolate B9 produces auxin as deduced by liquid chromatography tandem-mass spectrometry analysis. Such fungal auxin is secreted and contributes directly to enhanced root and shoot development and overall plant growth in *Arabidopsis thaliana*. Furthermore, auxin production by *P. citrinum* likely involves more than one tryptophan-dependent pathway. Using auxin biosynthesis inhibitor L-Kynurenine, we show that the indole-3-pyruvate pathway might be one of the key biosynthetic routes involved in such auxin production. Confocal microscopy of the *DR5rev:GFP* Arabidopsis reporter line helped demonstrate that *P. citrunum* B9-derived auxin is biologically active and is able to significantly enhance auxin signaling in roots during such improved root growth and plant development. Furthermore, the phenotypic growth defects arising from impaired auxin signaling in Arabidopsis *taa1* mutant or upon L-Kynurenine treatment of wild-type Arabidopsis seedlings could be significantly alleviated by fungus B9-derived auxin, thus suggesting its positive role in plant growth promotion. Collectively, our results provide clear evidence that the production of auxin is one of the main mechanisms involved in induction of the beneficial plant growth by *P. citrinum.*

## 1 Introduction

The Rhizosphere, the soil zone in the immediate vicinity (1–10 mm) of the roots, serves as the direct interface for the dynamic interplay between the soil, roots, and millions of microbial inhabitants (Bever et al., 2012; Ofek-Lalzar et al., 2014), that are collectively referred to as rhizosphere microbiota. It is now widely accepted that rhizosphere microbiota contributes to plant health, fitness, and performance under various environmental conditions. Importantly, they have the ability to enable and/or maintain water and nutrient status (Mohanram and Kumar, 2019; Pantigoso et al., 2022), provide tolerance to stresses (Khan et al., 2021), and improve resistance against pathogenic infection in host plants (Li et al., 2021; Khanna et al., 2022). Knowledge about the fungal symbiotic associations in the rhizosphere has been fundamental in the research of these synergistic modes of action and the search for bio-fertilizers in agriculture. In recent years, studies focusing on the fungal communities have characterized numerous root-associated fungi and their beneficial effects on plants and the ecological relevance of these interactions in nature (van der Heijden et al., 2015; Hiruma et al., 2016; Almario et al., 2017).

Further investigations have verified that increased phytohormone content and signaling in plants is one of the direct benefits of such interactions with rhizosphere fungi. Fungus-derived phytohormones, such as auxin, gibberellins, and cytokinins, are critical regulators involved in many aspects of beneficial associations in the plant–fungal systems (Khan et al., 2008; Vadassery et al., 2008; Hamayun et al., 2017; Pons et al., 2020), directly connected to greater plant biomass and higher growth rates.

Auxin is a pivotal phytohormone involved in almost every aspect of plant growth and development, including tropism, apical dominance, cell division and elongation, and the initiation of root formation (Péret et al., 2009; Gomes and Scortecci, 2021). Based on current fungal microbiota studies, indole-3-acetic acid (IAA) is the main active form of auxin produced by rhizosphere fungi for plant growth promotion. For example, the presence of IAA and auxin-related compounds (e.g., indole-3-acetaldehyde and indole-3-ethanol) are found in gnotobiotic *Trichoderma virens* culture filtrate. Detailed analysis of these compounds has revealed the efficacy of *T. virens-*derived IAA in the activation of auxin signaling, modulation of root system architecture, and the rescue of root hair defective phenotype in Arabidopsis lines (Contreras-Cornejo et al., 2009). *Piriformospora indica* is a well-known plant growth-promoting fungus with a broad host range. Importantly, *P. indica* produces IAA in liquid culture and such fungal auxin is involved in the reprogramming of the root architecture, leading to highly branched root systems in *Arabidopsis thaliana* (Sirrenberg et al., 2007). Furthermore, the production of IAA is also reported for the fungal endophytes *Paecilomyces formosus* LHL10 (Khan et al., 2012), *Phoma glomerata* LWL2, and *Penicillium* sp. LWL3 (Waqas et al., 2012). The beneficial interactions of these phytohormone-secreting fungal strains with the host crop species relieve adverse effects and support plant growth during stress conditions too.

In the past few years, an increasing number of research studies have been devoted to understanding the function of fungal microbiota in agriculture productivity. The use of biological inoculum in the agricultural sector would have greater biosafety, less environmental hazards, and higher efficiency. However, the theme in biological fertilizers based on these fungal products is still underexplored, especially for the mechanisms of plant growth promotion, although these associations with plants are as important as mycorrhizal symbiosis in nature. *Penicillium citrinum* isolate B9 is a successful example of plant growth-promoting fungi (PGPF), which was initially characterized from the rhizosphere of barley (*Hordeum vulgare*) and was found to promote the growth in Choy Sum (*Brassica rapa* var. *parachinensis*) under soil conditions (Gu et al., 2023). This fungal isolate is capable of producing bioactive gibberellins (GA_1_ and GA_3_), and the *P. citrinum* B9 exudates effectively enhance shoot biomass increase when provided exogenously to Arabidopsis plants. Interestingly, *P. citrinum* B9 inoculation also caused changes in the root system architecture in Choy Sum plants by enhancing root biomass and increasing root length. These modulations allow host plants to better adapt to the spatiotemporal heterogeneity of resource availability in soils. On the basis of the paramount importance of auxin on root development, we hypothesize the auxin production by *P. citrinum* B9 and its positive role in root growth regulation.

In this study, we present an Arabidopsis-based model system in which the PGPF strain *P. citrinum* isolate B9 was used to promote and improve root growth and architecture. The primary root elongation and lateral root branching phenotypes were found in plants inoculated with mycelia plugs of *P. citrinum* B9. A targeted analysis by liquid chromatography-mass spectrometry (LC-MS) of *P. citrinum* B9 culture filtrate or exudates revealed the presence of IAA, thus suggesting that *P. citrinum* B9 can produce and release this phytohormone into the host environment during mycelial growth. More importantly, the activity of *DR5rev:GFP* auxin reporter was significantly increased upon exposure to either *P. citrinum* B9 mycelia and/or the exudates thereof. These new findings provide evidence for the production of IAA by *P. citrinum* B9, and the growth-promoting effect of fungus-derived IAA in enhanced root growth in the host plants.

## 2 Experimental procedures

### 2.1 Fungus, plant material, and growth conditions

*P. citrinum* strain B9 stored as filter paper stocks was revived on Potato dextrose agar medium (PDA; VWR, Singapore), and fresh mycelial blocks from the hyphal margins were used for sub-culturing. Strain B9 was routinely cultivated in prune juice agar (PA) medium (Gu et al., 2023) or PDA plates while potato dextrose broth (PDB; VWR, Singapore) was used for culture filtrate extraction and IAA estimation. The plates were incubated at 28°C in the dark for 7 days.

Seedlings of *A. thaliana* ecotype *Columbia* (Col-0) wild type, *taa1* mutant line, and the transgenic line expressing the auxin reporter *DR5rev:GFP* (Benková et al., 2003; Friml et al., 2003) were used. *Arabidopsis* seeds were generally stored at 4°C in the dark to allow stratification and were surface sterilized by soaking in 70% ethanol for 5 min, treated with 10% commercial bleach for 2 min followed by five times rinsing in sterile distilled water. Seeds were germinated on plates containing full Murashige and Skoog basal salts (Sigma-Aldrich; no. M5524) with 1% sucrose and 1% agar (MS-agar). Plates with germinating seeds were incubated and grown vertically in a plant growth chamber AR95L (Percival Scientific, USA) under long-day conditions (16-h/8-h light/dark photoperiod) at 22°C with a relative humidity of 60% and 120 μmol m^−2^ s^−1^ light intensity.

### 2.2 Chemicals and standards

Certified standards of IAA, auxin biosynthesis inhibitor L-Kynurenine, and L-tryptophan (Trp) were purchased from Sigma-Aldrich (Darmstadt, Germany). Standard stock solution (1,000 μg/mL) of IAA was prepared in methanol and stored at −20°C in the dark. Stock solution was used to prepare working standard solutions for analytical experiments. For L-Kynurenine, a 1-mM stock solution was prepared in DMSO, which also served as a solvent only or mock control in parallel. Trp dissolved in distilled water (1 g/mL stock solution) was added to PDB at the indicated final concentration. Propidium iodide was purchased from Invitrogen (Carlsbad, USA). Stock solution (1 mg/mL) was prepared in distilled water with 10 μg/mL propidium iodide used as the working concentration for plant cell wall visualization. For LC-MS analysis, acetonitrile with 0.1% formic acid (Optima LC-MS grade) and methanol (Optima LC-MS grade) were obtained from Fluka Honeywell (Charlotte, NC, USA). Milli-Q water was used for the preparation of the mobile phase (Millipore, Burlington, MA, USA).

### 2.3 Plant–fungus co-cultivation

A plant–fungus interaction was fostered on a sucrose-free MS agar medium to assess the plant growth promotion by *P. citrinum* B9. Seven-day-old seedlings were transferred to sucrose-free MS agar medium (square dishes 17 cm × 17 cm) with or without mycelial plugs of B9 grown in PDA for 4 days, by locating their primary roots a few centimeters above the fungal inoculum and grown for another 7 days. For diffusible compounds (IAA), 7-day-old *Arabidopsis* seedlings were transferred to the vertical split-agar plates prepared with sucrose-free MS agar medium, which was segmented horizontally into the shoot and root sections and inoculated with B9 for 7 days in the root region supplemented with or without the inhibitor L-Kynurenine at the required concentration (Meier et al., 2020). Seven days after the treatment, the media plates were scanned using the EPSON V330 photo scanner, and the root length and area were measured using ImageJ software as previously described (Liu et al., 2018).

### 2.4 Preparation of culture-free extract for the detection of fungal auxin

For IAA extraction, fresh mycelial plugs of *P. citrinum* B9 grown in PDA or PA for 4 days were inoculated into PDB supplemented with or without 1 mg/mL Trp and/or L-Kynurenine. The flasks were incubated at 28°C, 180 rpm on an orbital shaker in the dark for the prescribed period. Culture supernatant was collected by filtering through four layers of Miracloth and centrifuged at 10,000 rpm for 10 min. Such cell-free extract was then used for the estimation of IAA using LC-MS analysis. For the estimation of IAA, the cell-free extract was then passed through a sterile filter membrane (0.2 μm), and the resulting extract was vacuum-dried by a CentriVap concentrator. The samples were reconstituted in 80% methanol (v/v) before analysis.

### 2.5 Liquid chromatography-mass spectrometry analysis of fungal IAA

LC-MS of the analyzed samples was acquired on an Agilent 1290 Infinity II coupled to an Agilent 6400 series Triple Quadrupole (Agilent, Santa Clara, CA, USA). The ultrahigh-performance liquid chromatography (UHPLC) system Agilent 6490 was equipped with a Kinetex C18 column (100-mm length, 2.1-mm inner diameter, 1.7-μm particle size, and 100 Å, Phenomenex, Torrance, CA, USA) heated at 35°C and the auto-sampler temperature was set at 4°C.

For the detection of IAA, 10 µL of fungal culture filtrate extracts was injected and separation was performed at a constant flow rate of 0.3 mL/min, in a gradient of Solvent A (water acidified with 0.1% formic acid) and B (acetonitrile acidified with 0.1% formic acid). The gradient program was as follows: 5% B (0–1 min); 5%–100% B (1–10.5 min); 100% B (10.5–13.4 min); re-equilibration from 100% to 5% B (13.4–13.5 min); and reconditioned with 5% B (13.5–16.5 min). Electrospray ionization was performed in negative ion mode in scheduled Multiple Reaction Monitoring (MRM) modes, with the following source parameters: a drying gas (N_2_) temperature of 250°C with a flow of 12 L/min, a nebulizer gas pressure of 35 psi, a sheath gas temperature of 350°C with a flow of 11 L/min, and a capillary voltage of 4,000 V. Data processing was performed using the associated MassHunter software B.10.00.

### 2.6 Confocal microscopy and imaging

In order to investigate the impact of fungal inoculation on the distribution of auxin maxima in roots, seedlings of *DR5rev:GFP* Arabidopsis reporter line were co-cultivated with B9 with or without the auxin inhibitor L-Kynurenine as mentioned above (Meier et al., 2020). DMSO was used as the mock control in parallel for each treatment. To assess the effect of *P. citrinum* B9-derived IAA on plant growth, a 100-μL aliquot of B9 exudates or culture filtrate was applied to the roots of Arabidopsis *DR5rev:GFP* line for 15 min. For imaging, roots of the *DR5rev:GFP* reporter line were stained with 10 μg/mL propidium iodide for 2 min, rinsed in water, and mounted on a glass slide with a coverslip. Imaging was done on a Leica TCS SP8 X inverted confocal system equipped with an HC Plan Apochromat 20×/0.75 CS2 Dry objective or a 63×/1.40 CS2 Oil objective. Green fluorescence (GFP) was excited at 488 nm and detected at 500–530 nm and red fluorescence (propidium iodide) was excited at 561 nm and detected at 600–700 nm. All parts of the system were under the control of the Leica Application Suite X software package (release version 3.5.5.19976).

### 2.7 Statistical analysis

Statistical analysis was carried out using GraphPad prism (GraphPad Software, San Diego, CA) and the values of the treatments were represented as mean with standard error. The significance of differences between the treatments was statistically evaluated using Student’s *t-*test and *post-hoc* Tukey’s HSD (honestly significant difference) test and significance was considered at a probability level of *p* < 0.05 (*), *p* < 0.01(**), and *p* < 0.001 (***).

## 3 Results

### 3.1 *Penicillium citrinum* isolate B9 promotes plant growth in *Arabidopsis thaliana*

**Figure 1:**
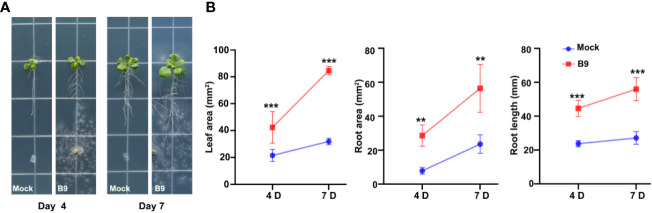
*P. citrinum* isolate B9 promotes root and shoot growth in *Arabidopsis*. **(A)** The representative images of *A. thaliana* Col-0 treated with *P. citrinum* in MS agar medium. *P. citrinum* B9 was inoculated as mycelial plugs while agar plugs lacking the fungus served as a mock or control sample. **(B)** Quantification of leaf area, root area, and root length from the *A. thaliana* Col-0 inoculated with *P. citrinum* B9. Data represent means ± SDs from three replicates consisting of five plants in each group. Differences were considered significant at a probability level of *p* < 0.01 (**) and *p* < 0.001 (***).

Our previous studies confirmed that *P. citrinum* isolate B9 promotes significant plant growth under both gnotobiotic and *in vivo* conditions in the Brassicaceae green leafy vegetable Choy Sum (Gu et al., 2023). *A. thaliana* serves as a model plant system to unravel the molecular and physiological responses of plants to microbial inoculation. Here, we started with co-cultivation experiments using MS agar medium initially to study the responses of *A. thaliana* to *P. citrinum* B9 using plant–fungal interaction assays (**Figure 1A**). Inoculation of B9 increased the primary root growth and the lateral root formation that ultimately resulted in a threefold increase in the root area of *Arabidopsis* wild-type (WT) Col-0 seedlings at 7 days of growth (**Figure 1B**). Inoculation of B9 further promoted a twofold increase in leaf area, together with root growth correlating to a doubling of the total fresh weight of inoculated plants (**Figure 1B**).

### 3.2 Using LC-MS to detect and analyze IAA produced by *P. citrinum* B9

**Figure 2:**
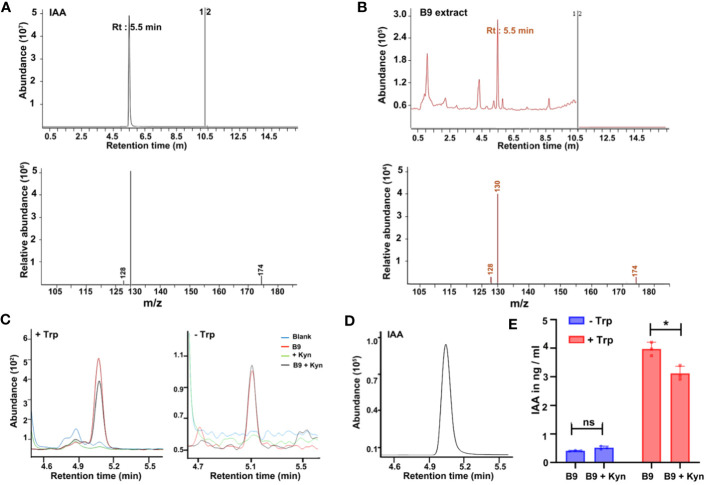
Auxin/IAA production by the *P. citrinum* B9 strain. **(A)** LC-MS chromatograms of IAA standard and tandem MS/MS spectrum of IAA standard. The fragmentation pattern of IAA indicates the *m/z* transition 174 > 130. **(B)** LC-MS chromatograms of B9 CF and tandem MS/MS spectrum of putative IAA. The fragmentation pattern of putative IAA indicates the precursor ion and product ion (*m/z* transition 174 > 130). **(C)** Chromatograms of IAA in the negative mode in *P. citrinum* B9 culture filtrate with (left) or without (right) Trp and/or in the presence of L-Kynurenine (Kyn; 50 μM) and **(D)** IAA external standard (1 μg/mL). *m/z* transition for IAA is 174 > 130. **(E)** Quantification of IAA in PDB and *P. citrinum* B9 culture filtrate. Trp (1 mg/mL) and/or L-Kynurenine (Kyn; 50 μM) was added to the PDB medium for the indicated treatment. Data represent means ± SD from three replicates consisting of three *P. citrinum* B9 exudates or culture filtrates in each group. Differences were considered significant at a probability level of *p* < 0.05 (*) based on ANOVA/Tukey’s HSD test.

Plant growth promotion by microorganisms is often attributed to the production of secondary metabolites, particularly the phytohormones such as auxins, cytokinins, and gibberellins, of which the IAA is the main auxin implicated in the regulation of cell division and elongation and the remodeling of root architecture in plants. Since *P. citrinum* B9 inoculation improved the root architecture and the growth of *Arabidopsis*, we reasoned whether this beneficial fungus produces bioactive auxin as a regulatory component that aids in root and/or plant growth promotion. Initially, we started with an analysis of the gnotobiotic culture filtrate of *P. citrinum* B9 by LC-MS together with the IAA standard (**Figure 2**). The MRM mode was used to assess the direct comparison of retention time associated with precursor-to-product ion *m/z* transition signals between the IAA standard (1 µg/ml / mL) and the corresponding compound(s) in the fungal exudate samples. Compound identification was confirmed through a product ion scan of the IAA standard and the corresponding fungal metabolite(s). IAA in *P. citrinum* B9 culture filtrate was determined at a retention time of 5.54 min in extracted single ion chromatogram with a precursor ion of *m/z* 174 [M-H] (negative ionization) and a product ion of *m/z* 130 (**Figure 2B**), which perfectly matched the results from the IAA standard.

### 3.3 IAA production by the *P. citrinum* B9 strain

As the LC-MS analysis confirmed that B9 unequivocally produces IAA and secretes it in the culture medium, we next analyzed the time course of IAA production in this beneficial fungal isolate. The main precursor for IAA synthesis is Trp and reports thus far have implied that IAA biosynthesis mostly occurs via the indole-3-acetamide (IAM) and indole-3-pyruvate (IPA) pathways in fungi while only a few species employ alternate or multiple pathways (Numponsak et al., 2018) for such auxin biosynthesis. Nevertheless, both Trp-dependent and Trp-independent IAA biosynthetic pathways coexist in plants and microbes (Fu et al., 2015). However, in the absence of Trp, IAA produced by B9 was in negligible amounts whereas, upon addition of Trp, there was a tremendous hike in the production of IAA as detected by LC-MS analyses (**Figures 2C–E**). Furthermore, when L-Kynurenine, an IPA pathway inhibitor that selectively targets TAA1/TAR (Trp aminotransferase of the TAA/TAR family) and effectively suppresses auxin biosynthesis (He et al., 2011; Won et al., 2011), was added to the growth medium along with Trp, there was a significant reduction in the auxin. However, L-Kynurenine had no effect on IAA production in *P. citrinum* B9 grown without Trp (**Figures 2C–E**). In addition, we used quantitative spectrophotometric assays using the Salkowski reagent to determine the total indolic compounds in the cell-free supernatant to analyze the temporal profile of IAA production in *P. citrinum* B9 (Gordon and Weber, 1951; Tarroum et al., 2022). Consistent with LC-MS analysis data, the addition of Trp led to increased IAA production in B9, whereas treatment with the auxin inhibitor significantly decreased the IAA production and secretion in *P. citrinum* B9 supplemented with Trp, and there was no significant difference in B9 growth *per se* in the absence of Trp, nor with or without L-Kynurenine (**Supplementary Figure S1A**). In general, the fungal IAA production reached a maximum at 6 days of B9 growth and declined at 10 days of culture when analyzed periodically (**Supplementary Figure S1B**). Taken together, these results revealed that IAA biosynthesis occurs in *P. citrinum* B9 via the Trp-dependent pathway and since L-Kynurenine treatment was able to reduce but not completely abolish the IAA levels, we further inferred that multiple pathways might be involved in auxin biosynthesis in *P. citrinum* B9 in addition to the partly shared IPA pathway that exists in Arabidopsis.

### 3.4 *P. citrinum*-derived IAA is bioactive and induces the auxin response in roots

**Figure 3:**
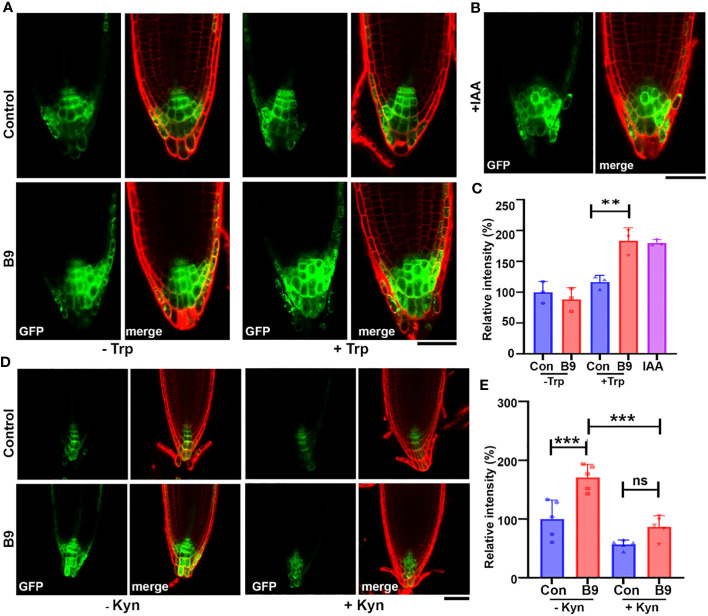
*P. citrinum* B9-derived IAA is bioactive and modulates the auxin accumulation and distribution in the host roots. **(A)** The symbiont *P. citrinum* B9 induces IAA in Arabidopsis roots. The representative confocal images of the DR5rev:GFP auxin reporter activities in the MS agar plates exposed to PDB and B9 culture filtrate supplemented with or without Trp (1 mg/mL) and **(B)** IAA (1 μg/mL), respectively. Roots/cell walls were stained with propidium iodide. Scale bar, 50 μm. **(C)** Quantification of the DR5rev:GFP epifluorescence intensity in the root tips. A total of three seedlings were imaged for each treatment (Student’s *t*-test). **(D)** The representative confocal images of the DR5rev:GFP auxin reporter in the MS agar medium supplemented with L-Kynurenine (Kyn; 5 μM) and inoculated with *P. citrinum* B9. *P. citrinum* B9 was inoculated as mycelial plugs while control was inoculated with medium plugs without the fungal mycelia/conidia. Roots/cell walls were outlined by staining with propidium iodide. Scale bar, 50 μm. **(E)** Quantification of the DR5rev:GFP fluorescence intensities in the root tips. A total of five seedlings were imaged for each treatment. Data represent means ± SDs from three replicates. Differences were considered significant at a probability level of *p* < 0.01 (**) and *p* < 0.001 (***). Statistical significance was derived using ANOVA/Tukey’s HSD test.

After confirming the IAA production in *P. citrinum* B9, we were interested to assess the growth-promoting effect of B9-derived auxin on root growth and its relationship with host auxin signaling in *A. thaliana*. To check if *P. citrinum* can alter auxin-regulated gene expression, we used seedlings of the auxin biosensor *DR5rev:GFP* containing an auxin-inducible marker gene and measured the response to inoculation with culture filtrate or exudates of *P. citrinum* B9 grown in the presence (B9-CF-Trp) or absence of Trp (B9-CF). *DR5rev:GFP* expression was strictly restricted to the center of the meristematic region in control plants and despite the fact that B9-CF contained a low amount of IAA, its exogenous addition failed to induce the *DR5rev:GFP* activity and the auxin distribution pattern was similar to the uninoculated control (**Figure 3A**). This might be due to negligible amounts of IAA that are not enough to trigger the auxin signaling. Nonetheless, the period of exposure should also be considered here, as auxin signaling was observed within a short period of exposure of the B9-CF to the root, which is not the case when plant roots are inoculated with B9. In contrast, inoculation with B9-CF-Trp enhanced the auxin signaling in *DR5rev:GFP*, and interestingly, in addition to enhancing the signaling in the meristematic region, the DR5rev:GFP signal also expanded into the columella (COL) and lateral root caps (LRC) of the reporter line that was similar to exogenous treatment with IAA (**Figure 3B**). As expected, Trp alone did not show any effect on DR5rev:GFP, implying that *P. citrinum* B9-derived IAA produced upon supplementation with Trp plays a role in modulating auxin signaling in the host plants. In parallel with the distribution of auxin, the DR5rev:GFP epifluorescence further remained comparable under the supply of exogenous IAA and B9-CF-Trp (**Figure 3C**). We conclude that the presence and the form of *P. citrinum* B9-derived IAA are most likely biologically active in the auxin signaling network of *A. thaliana*.

### 3.5 *P. citrinum* B9 suppresses the phenotypic defects associated with auxin deficiency in *Arabidopsis*

The enhanced *DR5rev:GFP* activity in the auxin biosensor provides evidence for the bioactive functionality of *P. citrinum* B9-derived IAA. To define more precisely the impact of this beneficial fungus on the host auxin network, we used the inhibitor L-Kynurenine to distinguish between the effect of endogenous auxin and B9-derived IAA. Since L-Kynurenine inhibits IAA biosynthesis via the IPA pathway in Arabidopsis, we inoculated B9 to the *DR5rev:GFP* seedlings grown with and without the auxin inhibitor. Inoculation of B9 showed an increased level of expression in the primary root apex similar to the B9-CF-Trp enhancement of the *DR5rev:GFP* expression at the COL and LRC and the relative epifluorescence (**Figure 3D**). On the other hand, treatment with L-Kynurenine depleted the endogenous auxin biosynthesis in Arabidopsis, thus decreasing the *DR5rev:GFP* signals dramatically. However, *P. citrinum* B9 inoculation could restore this impaired auxin biosynthesis, and a higher level of concomitant *DR5rev:GFP* expression and relative GFP epifluorescence was observed in the root apex of B9 inoculated and L-Kynurenine-treated plants (**Figure 3D**). Confocal epifluorescence imaging clearly provided evidence that B9-derived IAA is biologically active and can substitute or compensate for the loss of auxin signaling in the host.

**Figure 4:**
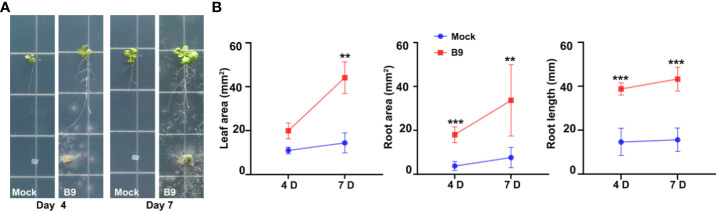
Mycobiont *P. citrinum* B9-derived IAA is functional and suppresses auxin-deficiency phenotypes in Arabidopsis roots. **(A)** The representative images of *A. thaliana taa1* mutant line treated with *P. citrinum* B9 in MS agar medium. B9 was inoculated as mycelial plugs while control refers to medium plugs in the absence of the fungal mycelia/conidia. **(B)** Quantification of leaf area, root area, and root length at the indicated time in the *A. thaliana taa1* mutant line in the absence (Mock) or presence of B9. Data represent means ± SDs from three replicates consisting of five plants in each group. Differences were considered significant at a probability level of *p* < 0.01 (**) and *p* < 0.001 (***).

Nevertheless, to assess whether the *P. citrinum* B9-derived IAA can simulate endogenous auxin and restore growth and development in plants lacking auxin, we designed a co-cultivation experiment with the Arabidopsis *taa1* mutant and observed the growth-promoting effect, if any, of *P. citrinum* B9 (**Figure 4**) therein. TAA1 is involved in the biosynthesis of IPA, a major precursor for auxin production. Defects in auxin biosynthesis lead to dwarfism and abnormal root development (e.g., shorter primary root and fewer lateral roots) in the *taa1* mutant plants (Stepanova et al., 2008). Interestingly, the alleviation of phenotypic defects associated with such auxin-deplete conditions was found in the *taa1* mutant plants upon B9 inoculation, thus indicating the production and functionality of fungal auxin during host interaction and its beneficial effects on plant growth and development. A similar suppressive trend via fungal auxin was also found in the L-Kynurenine-treated *A. thaliana* Col-0 seedlings. The inhibitory effect of L-Kynurenine in root growth was evident in split agar plate assays with Arabidopsis seedlings (**Supplementary Figure S2A**). Treatment with auxin inhibitor showed a negative impact on the overall root length and architecture, while inoculation with *P. citrinum* B9 could significantly suppress the defects imparted by L-Kynurenine (**Supplementary Figure S2B**). Notably, the treatment with such auxin inhibitor showed no discernible effect on fungal growth or colony morphology (**Supplementary Figure S3**). Taken together, these results confirm that the beneficial fungus *P. citrinum* B9-derived IAA is biologically relevant or functional to the host plant and enhances the host auxin signaling. In conclusion, our results unambiguously show that the plant growth-promoting mechanism of the *P. citrinum* isolate B9 includes the production of auxin/IAA to enhance the nascent auxin signaling and significantly induce growth in the host plant species.

## 4 Discussion

Beneficial rhizosphere microorganisms are considered to play a central role in the eco-centric organic-based modern agriculture scenario. PGPF account for 10%–44% of the total rhizosphere fungal populations, with their frequencies varying among the different host plant species. The *Penicillium* genus represents one of the most ubiquitous fungal species occurring in a diverse range of soils and has several known PGPF species that can colonize plant roots and provide growth enhancement (Hossain et al., 2007; Khan et al., 2008; Hossain et al., 2014; Babu et al., 2015). Aside from commonly occurring in soil, *P. citrinum* has also been reported to be an endophyte of various plants and remains the most frequently isolated species in the stem and roots of coffee plants (Posada et al., 2007), in roots of *Ixeris repenes* (Khan et al., 2008), and from leaves of *Catha edulis* (Mahmoud, 2000). The potential use in agriculture could be expanded if the plant growth-promoting mechanisms of these rhizosphere fungi were further understood. In this study, we show that the plant growth promotion imparted by B9, a PGPF having multiple plant growth-promoting characteristics and that improved the growth of the urban crop Choy Sum, is attributed mainly to its IAA-producing potential. Similar to the effect in Choy Sum, inoculation with *P. citrinum* B9 also improved the root system architecture in *Arabidopsis* in this study. The increased lateral root formation and leaf expansion in fungus B9 inoculated wild-type Col-0 (**Figure 1**) and *taa1* mutant line Arabidopsis (**Figure 4**) suggest that auxin/IAA is possibly involved in the plant growth-promoting mechanisms of this symbiont.

IAA is the most common indole-derived hormone of the auxin class, which can be produced by plants as well as some microorganisms, including bacteria (Cox et al., 2017; Gilbert et al., 2022) and fungi (Sirrenberg et al., 2007; Contreras-Cornejo et al., 2009; Khan et al., 2012; Waqas et al., 2012; Fu et al., 2015). PGPFs such as *Aspergillus* (Mehmood et al., 2019), *Penicillium* (Hasan, 2002; Waqas et al., 2012), and *Trichoderma* (Contreras-Cornejo et al., 2009) have been characterized to produce IAA and contribute to plant growth and development. In plants, IAA is a signaling molecule involved in the coordination of growth and development. It is essential for tissue differentiation, response to light and gravity, and the lateral branching of shoots and roots. In plant–microbe interactions, microbial IAA and/or its analogs produced by phytopathogens act as the virulence factor(s), disturbing the homeostasis in plants and promoting disease development (Spaepen and Vanderleyden, 2011; Patten et al., 2013; Duca et al., 2014; Kunkel and Harper, 2018). However, microbial IAA is also a growth stimulator, which has become an area of great interest in fungal ecology in recent years. In this study, we detected the presence of IAA in *P. citrinum* B9 growth medium by the LC-MS system. Such fungal IAA production and secretion increased upon the supplementation of the growth medium with Trp (**Figure 2**). In a few species of *Penicillium*, although the production of IAA was reported to be one of the plant growth-promoting mechanisms (Hasan, 2002; Waqas et al., 2012), clear evidence for the role of fungal-produced IAA in plant growth promotion or the pathway for IAA biosynthesis is lagging behind. Interestingly, a reduced IAA production with L-Kynurenine addition to the B9-CF-Trp (**Figure 2**), but not complete inhibition, suggests that IAA production in *P. citrinum* B9 may involve multiple Trp-dependent pathways, including the IPA pathway. However, to fully elucidate the IAA biosynthesis in B9, metabolic analysis by the LC-MS system is necessary to explore the intermediates in both IPA and other Trp-dependent IAA biosynthetic pathways in future experiments.

Microbial auxin should not only be synthesized and secreted but must also enter host root cells in sufficient quantities to alter normal plant growth and development to have a role in interfering with developmental pathways and modulating auxin biosynthesis and/or signaling in the host (Sukumar et al., 2012). Few studies have demonstrated that microbial auxin directly or indirectly modulates host auxin homeostasis that is achieved through synthesis, conjugation, degradation, transport, and signaling of auxin (Sukumar et al., 2012). In our study, we showed that the enhanced expression of auxin-induced marker *DR5rev:GFP* in the Arabidopsis reporter line when co-cultured with B9 mimicked the exogenous addition of IAA (**Figure 3**). Furthermore, the *P. citrinum* B9-culture filtrate also showed the enhancement of auxin signaling that further confirmed that *P. citrinum* B9-derived IAA is biogically active and, in addition to acting as a molecular signal, has a precise role in interfering or altering auxin synthesis or signaling in host roots.

From our results, it is clear that *P. citrinum* B9 produces IAA that is likely sensed by the host roots and involved in enhanced plant root development. However, an intriguing question now is whether such microbial auxin transportation has a role in plant–fungus interactions. Polar auxin transport is an active process for auxin movement between cells in a directional and polarized manner (Muday and DeLong, 2001) that is attributed to the AUX1/LUX family of auxin influx carriers, the PIN family of the auxin efflux carrier, and the PGP/ABCB family of auxin transporter (Peer et al., 2011) in *A. thaliana*. These proteins coordinately regulate local auxin gradients and ultimately determine the entire architecture of a plant.

Furthermore, WEI8/SAV3/TAA1, the tryptophan aminotransferase, regulates auxin biosynthesis in response to ethylene, and during shade avoidance too (Stepanova et al., 2008; Tao et al., 2008). Such hormonal crosstalk centered on auxin signaling is also critical for plant growth and development during stress conditions. Evidence for ethylene-triggered regulation of auxin signaling (DR5rev:GFP) in the root tips has been demonstrated previously (Stepanova et al., 2008), along with the role of auxin/TAA1 in enabling shade avoidance in Arabidopsis (Tao et al., 2008). More importantly, such a regulated increase in auxin signaling/production by ethylene plays a direct role in downstream developmental responses (Stepanova et al., 2008; He et al., 2011). However, the regulatory effect of ethylene on plant growth is subject to intricate control via several environmental cues. For instance, ethylene is an important hormone released by plants during biotic- and abiotic stress conditions (Berrocal-Lobo et al., 2002; Lorenzo et al., 2003; Berrocal-Lobo and Molina, 2004). Although the activation of ethylene signaling has been found during symbiotic interactions, the major role of this hormone is to recruit plant defense, i.e., limiting the growth of fungal pathogens in root tissues and maintaining plant fitness (Camehl et al., 2010). Although less likely, it remains to be seen whether ethylene contributes to the beneficial growth promotion effect enabled by the *P. citrinum*–plant symbiosis. Likewise, the molecular basis of the complex regulatory network of auxin signaling and its physiological effectors remains to be investigated in our mycobiont system. In the *P. citrinum*–*A. thaliana* interactions, the fungus-derived IAA likely serves as an environmental stimulus that regulates the expression and localization of these proteins to sense/transport fungal IAA for beneficial effects. In the future, our study will focus on investigating the response of Arabidopsis auxin transport mutants to *P. citrinum* mycelia and/or exudates to better understand the role of fungal IAA in the behavior between beneficial fungi and plants in the rhizosphere. In conclusion, with the reliance on urban farming, particularly organic farming, the feasibility of technology using naturally occurring PGPF relies on better-quality inoculants that satisfy the needs of changing conditions. *P. citrinum*-based bio-inoculants could thus be explored further to study the synergistic interactions with other urban or traditional crops for a better understanding of their ecological fitness and/or mechanism(s) of action in agricultural productivity.

## Data availability statement

The original contributions presented in the study are included in the article/**Supplementary Material.** Further inquiries can be directed to the corresponding author.

## Author contributions

C-YC, PS, and NN designed the experiments. C-YC and PS performed all the experiments. C-YC, PS, and NN interpreted and analyzed the data, compiled all the results, and co-wrote the manuscript. NN provided funding support and resources, and overall project management. All authors agree to the final submitted version of the manuscript.
