# Supplementary material for: Functional analysis of auxin derived from a symbiotic mycobiont
Source: Front Plant Sci. 2023 Sep 8;14:1216680. doi: 10.3389/fpls.2023.1216680 (PMC10515717; doi:10.3389/fpls.2023.1216680)

Supplementary Material

Functional analysis of auxin derived from a symbiotic mycobiont

Cheng-Yen Chen[1,†], Poonguzhali Selvaraj[1,†], Naweed I. Naqvi[1,2,*]

*** Correspondence:** Naweed I. Naqvi (naweed@tll.org.sg)

Supplementary Figure 1.

Auxin/IAA production by the P. citrinum B9 strain. (**A**) IAA detected in the cell free supernatant of B9 with and without the inhibitor and (**B**) the temporal profile of IAA production by B9 analyzed at different time points. The cultures were withdrawn at the indicated time points and the presence of indolic compounds detected using Salkowski reagent in a spectrophotometer at 530 nm. Data represents means ± SD from 3 replicates consisting of 3 P. citrinum B9 exudates or culture filtrates in each group. Differences were considered significant at a probability level of $P < 0.05$ (*) and $P < 0.001$ (***).

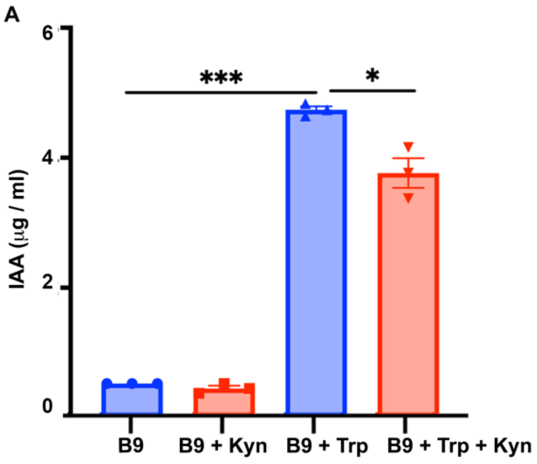

Supplementary Figure 2.

P. citrinum B9-derived IAA is critical for the development of higher-order root system architecture. (**A**) The representative images of *A. thaliana* Col-0 treated with L-Kynurenine (5 µM) and *P. citrinum* B9 in the split MS agar plates medium. B9 was inoculated as mycelial plugs while control was in the absence of the fungal mycelia/conidia. L-Kynurenine was exclusively supplemented in the root segment of split MS agar plates. (**B**) Quantification of leaf area, root area, and root length from the *A. thaliana* Col-0 treated with L-Kynurenine and inoculated with B9. Data represent means ± SDs from 3 replicates consisting of 6 plants in each group. Statistical analyses were carried out using ANOVA / Tukey's HSD test. Differences were considered significant at a probability level of $P < 0.05$ (*), $P < 0.01$ (**) and $P < 0.001$ (***).

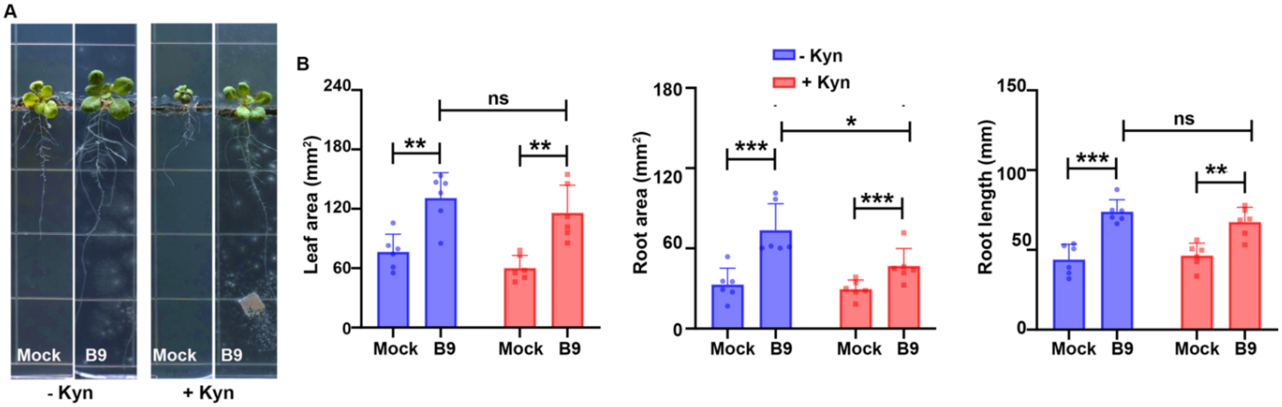

Supplementary Figure 3.

Hyphal growth of *P. citrinum* B9 is unaffected in the presence of Tryptophan (+Trp) or the auxin inhibitor L-Kynurenine (+ Kyn). *P. citrinum* B9 was grown in Prune agar or Murashige Skoog medium with Trp (1 mg/ml) or L-Kyn (1 μM) and incubated for 4 days at room temperature and resultant colonies observed for growth differences thereafter.

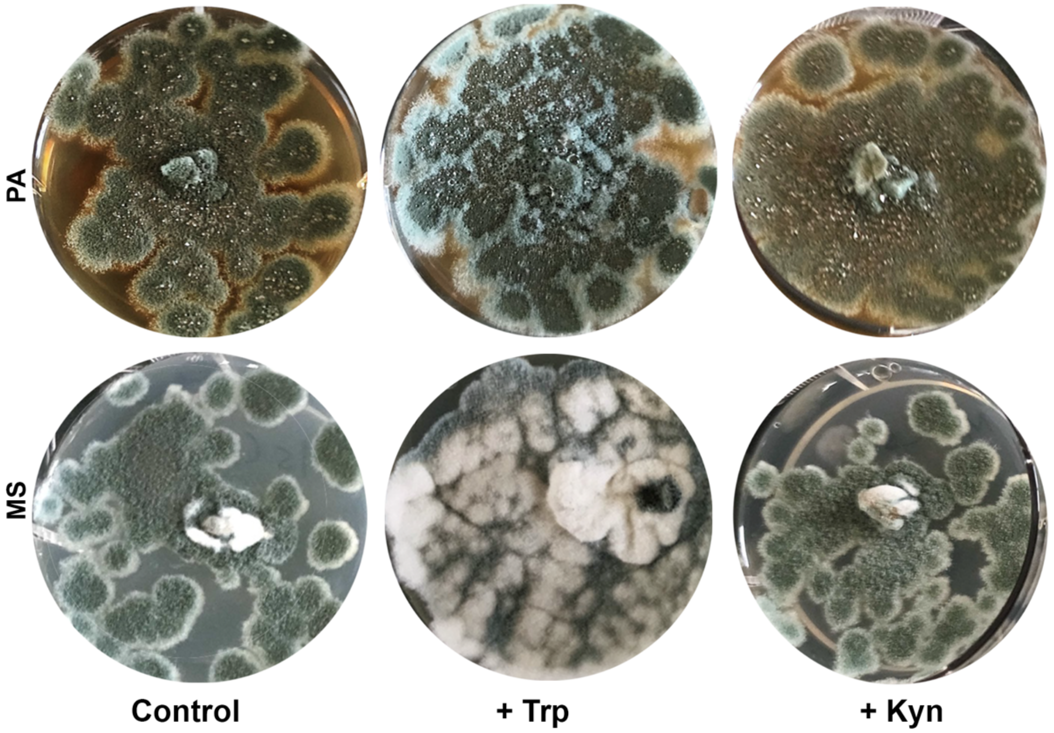

Supplement: Supplementary file 1 [file DataSheet_1.pdf]
